# Conformational Signatures of Preassembled and Active Complexes of 5‑HT_7_ with the G_s_ Protein

**DOI:** 10.1021/acs.jcim.5c01698

**Published:** 2025-10-24

**Authors:** Zeenat Zara, Alessandro Nicoli, Ruiming He, Natalia Kulik, David Reha, Alexey Bondar, Antonella Di Pizio

**Affiliations:** † 28362 Leibniz Institute for Food Systems Biology at the Technical University of Munich, Freising 85354, Germany; ‡ Faculty of Science, University of South Bohemia in Ceske Budejovice, Branisovska 1760, Ceske Budejovice 370 05, Czech Republic; § Professorship for Chemoinformatics and Protein Modelling, TUM School of Life Sciences, Technical University of Munich, 85354 Freising, Germany; ∥ Center for Functional Protein Assemblies (CPA), Department Bioscience, TUM School of Natural Science, Technical University of Munich, Ernst-Otto-Fischer-Strasse 8, Garching 85748, Germany; ⊥ Laboratory of Photosynthesis, Centre Algatech, Institute of Microbiology of the Czech Academy of Sciences, Novohradská 237, Třeboň CZ-37981, Czech Republic; # IT4Innovations, VSB—Technical University of Ostrava, 17. Listopadu 2172/15, Ostrava-Poruba 708 00, Czech Republic; ¶ Institute of Plant Molecular Biology, Biology Centre of the Czech Academy of Sciences, Branisovska 1160/31, Ceske Budejovice 370 05, Czech Republic

## Abstract

5-Hydroxytryptamine receptor type 7 (5-HT_7_) receptor is a G protein-coupled receptor (GPCR) exhibiting noncanonical signaling properties. It has been shown that 5-HT_7_ can form stable inactive preassembled complexes with its cognate G_s_ protein. Structural determinants of such complex formation and the distinction between preassembled and intermediate activated complexes remain unknown. Here, we use molecular modeling and molecular dynamics simulations to determine and characterize the binding interface between this receptor and the G_s_ protein in both the active and preassembly complexes. Our results show key interaction patterns specific for the different states and pinpoint unique structural features distinguishing active, inactive, and preassembled states of the receptor.

## Graphical abstract

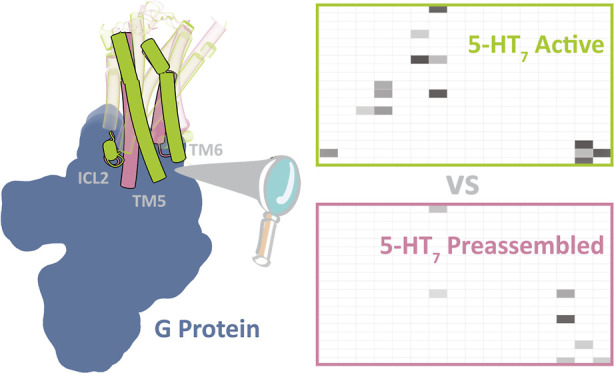

## 1 Introduction

The 5-hydroxytryptamine receptor type 7 (5-HT_7_) receptor is a signaling protein widely expressed in glial cells and neurons throughout the central nervous system, including the spinal cord, thalamus, hypothalamus, amygdala, and suprachiasmatic nucleus,^1–4^ where it is involved in many physiological activities, including the sleep cycle, circadian rhythm, rapid eye movement, thermoregulation, and memory.^1,5^ In the gastrointestinal tract, 5-HT_7_ is expressed in immune cells in lymphoid tissues, where it plays a role in the inflammation response.^6^ Dysregulation of 5-HT_7_ signaling may cause various pathological conditions, including neurodegenerative diseases, cognitive disorders, depression, and immune system-related diseases.^6,7^ Therefore, 5-HT_7_ represents an intriguing target for therapeutic applications.

5-HT_7_ is a member of the G protein-coupled receptor family (GPCR), a family of membrane proteins that are targeted by 34% of approved drugs.^8,9^ 5-HT_7_ belongs to the serotonin subfamily of class A GPCRs. Humans express 7 types of 5-HT receptors: 5-HT_1_ (5-HT_1A_, 5-HT_1B_, 5-HT_1C_, 5-HT_1D_, 5-HT_1E_, 5-HT_1F_), 5-HT_2_ (5-HT_2A_, 5-HT_2B_, 5-HT_2C_), 5-HT_3_, 5-HT_4_, 5-HT_5_, 5-HT_6_, and 5-HT_7_,^10^ which are all GPCRs with the exception of 5-HT_3_ that is an ionotropic receptor. GPCRs signal primarily through the activation of heterotrimeric G proteins composed of α, β, and γ subunits. Upon agonist binding, e.g., serotonin (5-hydroxytryptamine, 5-HT), to 5-HT receptors, the receptor undergoes conformational changes leading to the recruitment of a G protein.^11,12^ The activation of the G protein results in the dissociation of the G_α_ subunit from the G_βγ_ and subsequent attenuation of downstream pathways.^13^ The 16 human genes encoding G_α_ proteins are categorized into four major families: G_S_ (G_s_ and G_olf_), G_i/0_ (G_i1_, G_i2_, G_i3_, G_o_, G_z_, G_t1_, G_t2_, and G_gust_), G_q/11_ (G_q_, G_11_, G_14_, and G_15_), and G_12/13_ (G_12_ and G_13_). The 5-HT receptor subtypes vary in their primary coupling. 5-HT_1_ and 5-HT_5_ couple to G_i/0_, 5-HT_2_ to G_q/11_, and 5-HT_4_, 5-HT_6_, and 5-HT_7_ couple to the G_S_.^14^

In addition to the canonical G protein coupling, 5-HT_7_ and several other GPCRs can engage with G proteins in the inactive state.^15–24^ This mode of coupling is termed precoupling, preassembly, or inverse coupling. 5-HT_7_ forms a long-lasting inactive complex with the G_s_ protein,^18^ which has been proposed to downregulate the intrinsic basal activity of 5-HT_7_.^19,22^

The structure of 5-HT_7_ in the active state in complex with its downstream partner G_s_ protein has been released,^29^ however structural insights into the inactive state and preassembled complex are still lacking. Understanding the molecular basis of 5-HT_7_ signaling will be valuable for the design of precise modulators of this receptor. In this work, we characterized snapshots of the major conformational states of 5-HT_7_ by integrating molecular modeling and molecular dynamics (MD) simulations. MD simulations have been successful in revealing transient interactions and the inherent flexibility, such as breathing motions, of GPCRs, even over relatively short time scales.^25,26^ Our MD analysis enabled the identification of unique, state-specific contact patterns and conformational features that regulate the 5-HT_7_:G_s_ preassembled complex. This provides insights into the functional role of this state in the activation mechanism of 5-HT_7_.

## 2 Results and Discussion

Upon activation, 5-HT_7_ undergoes a conformational change from the inactive to the active state and interacts with the G_s_ protein heterotrimer (G_αβγ_). The formation of the fully activated complex catalyzes the exchange of guanosine diphosphate (GDP) for guanosine triphosphate (GTP) on the G_α_ subunit and subsequently activates downstream signaling pathways.^27^ In the inactive state, 5-HT_7_ can associate with the GDP-bound heterotrimeric G_s_ protein and form a stable complex.^18^ In our study, we combined molecular modeling and MD simulations for a comprehensive structural analysis of the active state of 5-HT_7_ in complex with the nucleotide-empty G_s_ protein, the inactive state of 5-HT_7_ and the preassembled 5-HT_7_:G_s_ complex (Figure 1).

**Figure 1:**
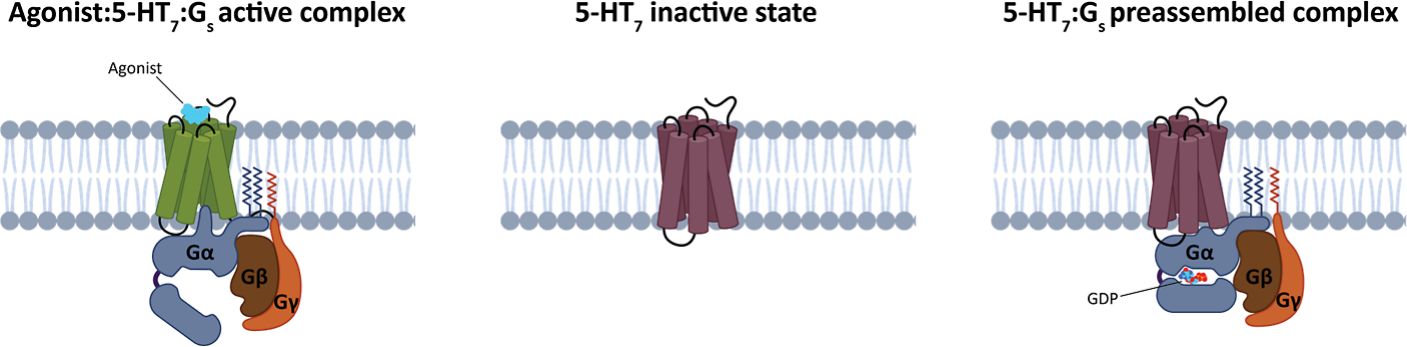
Schematic representation of the three states modeled and simulated in this study. 5-HT_7_ is colored in green in the active state and dark purple for the inactive state. The G protein subunits α, β, and γ are colored in blue, brown, and orange, respectively. The active state was simulated in the presence of the cocrystallized agonist, while the preassembled state in the presence of the guanosine diphosphate (GDP).

To facilitate structural analyses and comparisons, throughout this work, we use numbering systems and terminology specifically developed for GPCRs and G proteins. All GPCRs share a common structural architecture of seven transmembrane (7TM) α-helices connected by three intracellular loops (ICL1, ICL2, and ICL3) and three extracellular loops (ECL1, ECL2, ECL3).^11^ According to the Ballesteros–Weinstein (BW) numbering scheme, TM residues are numbered referring to the highest conserved residue of each TM as the position X.50 (X indicates helix number).^28^ The G_α_ subunit consists of two main domains: a Ras domain (RD) that is involved in the binding and hydrolysis of GTP and the helical domain (HD) that buries the GTP within the core of the protein (Figure S1). The RD is composed of six β (β1−β6) strands and five α-helices (H1–H5). The HD consists of six α-helices (HA–HF) that are inserted between H1 and β2 of the Ras domain. A αN helix (HN) is located before the Ras domain. Residues belonging to RD, HN, and HD are numbered according to their position in the secondary structure, e.g., the first residue of the α5 helix is residue position G.H5.01, where G represents the G_α_ subunit, G.H5 represents the α5 helix, and the 01 stands for the first residue of the α5 helix. Similarly, the position G.S6.03 represents the third residue of the β6 strand, where S6 reflects the β6 strand and 03 represents the third residue of this β6 strand. Loop regions are commonly named based on structured regions present before and after that loop, for example, the residue S84^G‑h1ha.20^ is a loop region between H1 and HA, and 20 stands for the 20th residue of this loop, whereas A48^G‑s1h1.02^ is a loop residue between β1 and H1 and the 02 is the second residue of this loop. In the case of loop regions, small letters are used to refer to the structured regions.

### 2.1 Preassembled and Active Complex of the 5-HT_7_ Receptor with the Heterotrimeric G_s_ Protein

The preassembled complex is formed between inactive 5-HT_7_ and inactive GDP-bound G_s_ protein (Figure 1). As the structure of 5-HT_7_ is experimentally available only in the active state,^29^ we modeled the 5-HT_7_ structure in the inactive state (Figure S2). We then modeled the preassembled complex between the inactive 5-HT_7_ with the refined inactive G_s_ protein bound to GDP, following a previous computational study on preassembled complexes of 5-HT_1A_ and other class A GPCRs.^30,31^ In the modeled preassembled complex, G.H5 is not deeply inserted into the intracellular side of 5-HT_7_ due to the inward position of TM6, which occludes the G protein binding site (Figure 2). The G.H5 has a pivotal role on GPCR activation, it fully extends inside the intracellular side of the GPCR and initiates allosteric conformational alterations in proximity to the nucleotide-binding pocket, resulting in GDP release and the opening of the HD.^32^ Instead, H5 in the preassembled complex has a different angle of tilt of the α-helix compared to its conformation in the G_s_ nucleotide free state (Figure 2). Because of these differences, the interface of the full active complex is much wider (4035 Å^2^) compared to the interface of the preassembled complex (3660 Å^2^) (Figure S3).

**Figure 2:**
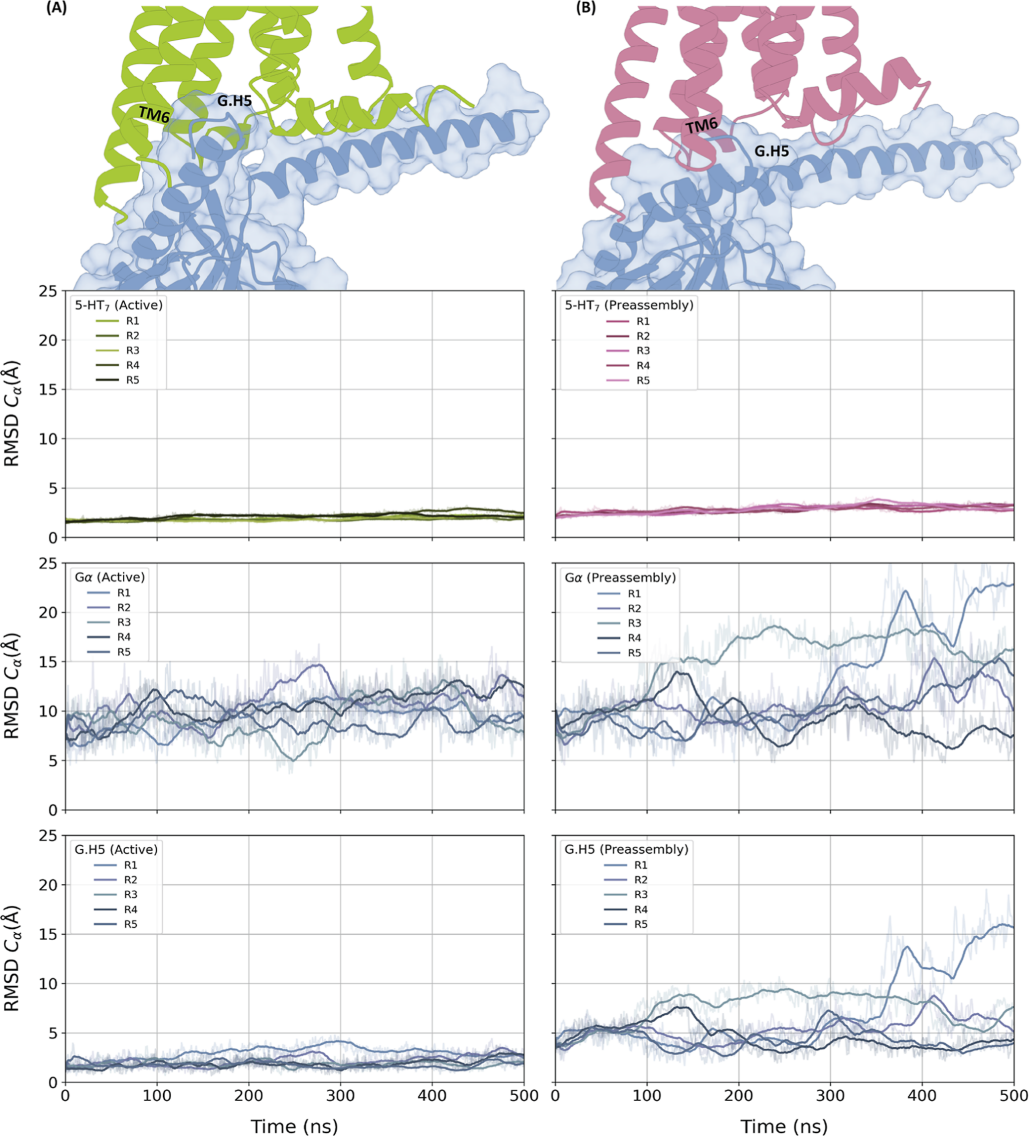
Flexibility of 5-HT_7_ and G_s_ during MD simulations. On the top, the representation of the interacting interfaces between 5-HT_7_ and G_s_ protein in the fully active (A) and preassembled complexes (B). 5-HT_7_ is colored in green in the active state and purple for the inactive state. The G protein α subunit is colored in blue. H5 of the G protein and TM6 of 5-HT_7_ are annotated. The electrostatic surfaces of the complexes are reported in Figure S3. Bottom panels report the root-mean-square deviation (RMSD) plots for 5-HT_7_, G_α_, and G.H5 for the active (A) and preassembled complexes (B). The carbon alpha atoms of 5-HT_7_ (excluding the ICL3 region) were used as the reference for the structural alignment. RMSD plots of all the domains are reported in Figure S4.

To explore the local flexibility of the fully active and preassembled complexes, we performed MD simulations. We ran five independent replicas of 500 ns for each system (total aggregate time of 5 μs). The Root Mean Square Deviation (RMSD, C_α_ of each chain vs the initial coordinates) analyses revealed that the complexes are quite stable, even in the modeled regions (Figure S4). To understand the stability of the complexes along the simulations, we monitored the movement of the G_s_ protein in relation to 5-HT_7_ (Figure 2). We observed that G_β_ and G_γ_ are more flexible in the preassembled complex than in the fully active complex, due to the local adjustment of the whole G_s_ protein toward the inactive 5-HT_7_ (Figure S4). This suggests that these domains are less involved in the interaction in the preassembled state. While the G_α_ protein is quite stable throughout the simulation period, we observed a higher flexibility in the region of the long unstructured loop (V63^G.h1ha.01^–S86^G‑h1ha.20^) in the HD for both complexes (as measured by the Root Mean-Square Fluctuation, RMSF, Figure S6). Zooming in on G.H5 (T369^G.H5.01^–L394^G.H5.26^), we observe relatively stable dynamic behavior throughout the simulation in the active complex (Figure 2). This is a consequence of the extensive surface contacts (Figure S3) and multiple interactions that firmly anchor G.H5 to 5-HT_7_. The G.H5 conformational space sampled in the active complex is more restricted than in the preassembled state (average RMSD is around 5 Å), where side chains continuously adjust throughout the simulation (Figure S7). Moreover, the fully active complex displayed higher peaks in the HB–HE region of the HD domain (E123^H.HB.01^–V184^H.HE.1^, Figure S6). This difference can be attributed to the fact that in the preassembled complex, the helical domain is tightly bound to the GDP and the RD domain, thereby reducing the overall local mobility.

### 2.2 Interaction Interfaces for the Fully Active and Preassembled Complexes

To understand the key contacts that stabilize the complexes, we monitored the interactions between 5-HT_7_ and the G_α_ protein subunit for both active and preassembled complexes. In Figure 3, we report the regions involved in the interaction.

**Figure 3:**
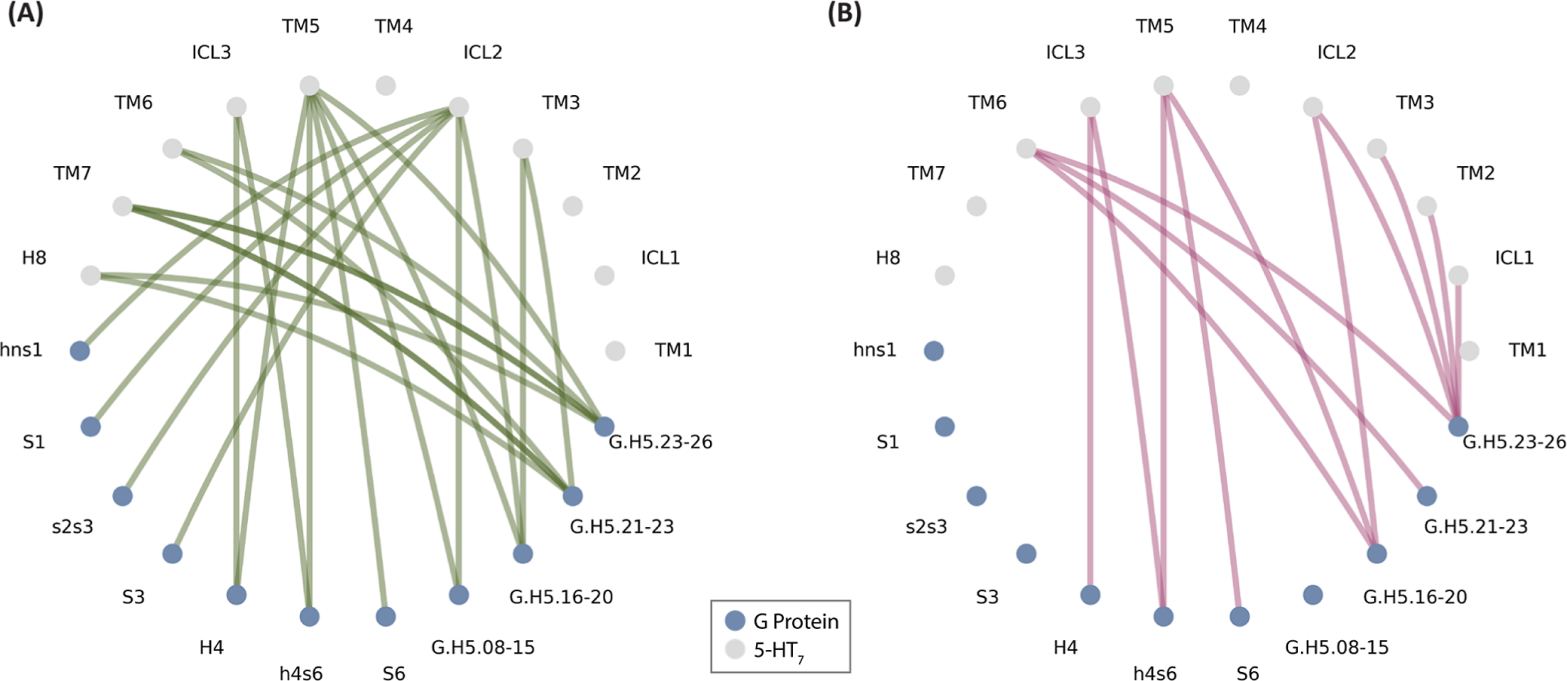
Flare plots representing the interactions between G_α_ and 5-HT_7_ domains in the active (A) and preassembled complex (B). The nodes are colored in blue and gray for the G protein and 5-HT_7_ domains, respectively. The edges are colored in green for the interactions in the active complex and in purple for the interactions in the preassembled complex.

The interacting residues in 5-HT_7_ include TM3, ICL2, TM5, TM6, ICL3, TM7, and H8 in the full active complex, whereas TM2, ICL2, TM5, ICL3, and TM6 in the preassembled complex. Therefore, several state-specific interactions are revealed. ICL1 and TM2 residues are involved in interactions in the preassembled complex but not in the fully active state. TM3 residues interact with different residues of H5 in the fully active complex with respect to the preassembled complex. ICL2 interacts exclusively with H5 in the preassembled complex, while, in the active complex, it establishes contacts with different regions of H5 in addition to HN, hns1, S1, S3, and s2s3. TM5 in the active complex establishes contacts with H4 that are absent in the preassembled complex. ICL3 is instead in contact with h4s6 and H4 in both complexes. TM6 residues interact with H5 residues; however, the interacting residues for active and preassembled complexes are different. TM7 and H8 are involved in interactions only in the active complex.

### 2.3 5-HT_7_ Specificity for the Preassembled Complex and Insights into the Activation Mechanism

Our work revealed several state-specific interactions, many of which emerge and/or become more significant during MD simulations. Figure 4 presents the patterns of interactions in the active (green contour) and preassembled (purple contour) complexes before (Figure S8) and after the simulations.

**Figure 4:**
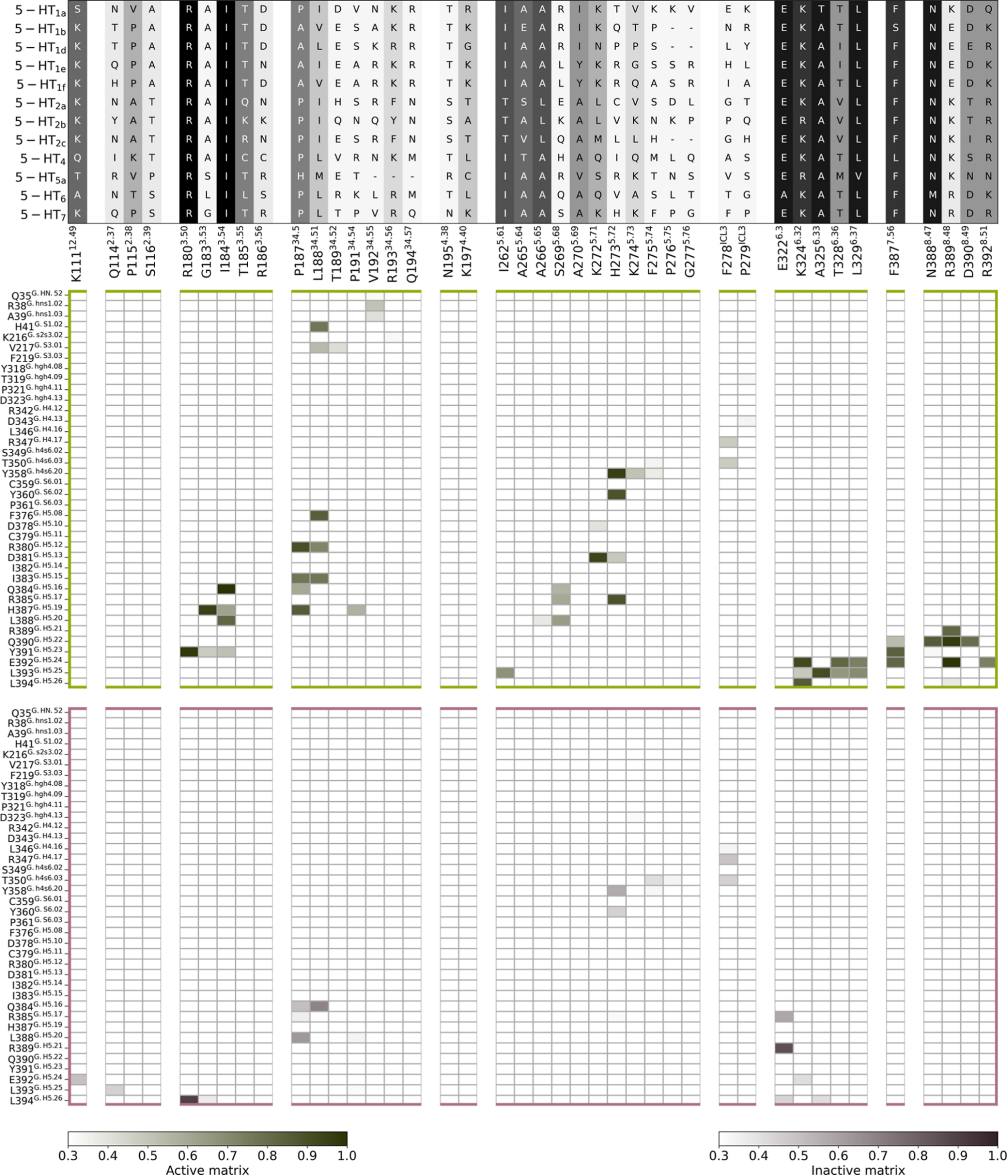
Interaction profiles of active (green framed) and preassembled (purple framed) 5-HT_7_ in complex with G_α_ during the MD simulations. 5-HT_7_ interacting residues are reported in the *x* axis and G protein residues in the *y* axis. Contacts in the structural models are reported in the matrix with green and dark-purple shades for active and preassembled according to the frequency of interactions in the simulations. On the top of the panel, the sequence alignment of 5-HT_7_ interacting residues to other 5-HT receptors is reported and colored with shades of gray. The matrices of the starting complexes are reported in Figure S8. The per-residue interaction frequencies between 5-HT_7_ and G_α_ are mapped onto the structural representation of the proteins in Figure S9.

The G.H5 serves as the primary interacting helix in both the active and preassembled complexes, but our analyses show that the increased protrusion of G_α_ within the intracellular side of 5-HT_7_ in the active state leads to a higher number of interactions of H5 with the receptor compared to the preassembled complex, impacting their local stability (Figure 2). The difference in interactions depends on the different conformations of H5 in the preassembled complex (Figure S7). The RMSD of C_α_ of G.H5 (residues T369^G.H5.01^–L394^G.H5.26^) between the initial active and preassembled complexes is 6.32 Å. Interestingly, the MD simulations reveal new interactions compared to the experimental structure of the active complex, demonstrating how MD analyses could complement experimental structural data (Figures 4, S8, and S9).^33^ Particularly, the H8 of 5-HT_7_ increased the number of residues in contact with H5, and ICL2 engaged and established new interactions with H5, HN, S1, the s2–s3 loop, and S3. On the other side, many interactions are maintained in all the MD replicas for the entire simulated time; e.g., the interactions between Y391^G.H5.23^–-R180^3.50^, H387^G.H5.19^–G183^3.53^, Q384^G.H5.16^–I184^3.54^, E392^G.H524^–K324^6.32^, and I383^H5.15^ with P187^34.50^ and L188^34.51^ are present in the simulations of the active complex with a frequency of 100%.

Although the structure of the preassembled complex is a model, we found that many of the interactions in the initial structure were retained during the simulations (i.e., H387^G.H5.19^–G183^3.53^, L346^G.H4.16^–F278^ICL3^, and also P187^34.50^ and P191^34.54^ with L388^G.H5.20^). However, new interactions that stabilize the complex appear during the simulations (Figures 4, S8). Specifically, we found that E322^6.30^ interacts with R385^G.H5.17^ and R389^G.H5.21^ residues and the conserved R180^3.50^ with the C-terminus of L394^G.H5.26^ (frequent interactions, >60%). The hydrophobic interactions established between the ICL2 of 5-HT_7_ and H5 also increase during the simulations.

Overall, the MD analyses proved to be a valuable tool for optimizing both the preassembled and active complexes. By closely examining key interactions, we identified positions that are highly conserved among all 5-HT receptors, as well as positions that are specific to the 5-HT_7_ receptor.

The identified conserved residues are known to play an important role in GPCR activation, which supports the hypothesis of a common activation mechanism for class A GPCRs.^34^ R180^3.50^ belongs to the conserved DRY motif and establishes a conserved ionic lock with E322^6.30^, which is also conserved in class A GPCRs, holding TM3 and TM6 together in the GPCR inactive state. Upon activation, R180^3.50^ swings to interact with the switch Y^5.58^ and Y^7.53^as well as with the G protein.^35^ Indeed, the interaction between R180^3.50^ and Y391^G.H5.23^ is present in our initial active structure and is maintained during simulations.

To analyze the dynamic behavior of these residues in the inactive state of 5-HT_7_, we run MD simulations (three replicas of 500 ns) for the inactive state model of 5-HT_7_ without the G_s_ partner. The RMSD profile of the apo receptor demonstrates overall stability over the course of the simulation (Figure S10). The ionic lock between R180^3.50^ and E322^6.30^, manually introduced in the initial model, is maintained during the simulations of apo inactive 5-HT_7_ (Figure S10). Instead, it dissociates within the initial 200 ns of all the preassembly replicas (Figure S11) to allow the cytoplasmic region to slightly open up and help G.H5 to form a novel set of interactions: the interactions between E322^6.30^ with R385^G.H5.17^ and R389^G.H5.21^. These interactions are not observed in the active complex nor in the initial preassembly complex but arise only during MD simulations of the preassembled complex.

Here, we propose a mechanism in which the change in interactions of TM3 from the inactive to active state passes through an intermediate conformation stabilized by the preassembled complex with G_s_. In this intermediate conformation, R180^3.50^ assumes a new conformation and engages in an ionic interaction with the negatively charged C-terminus of L394^G.H5.26^ (Figure 5A). Importantly, L394^G.H5.26^ is in close proximity to G183^3.53^, which is a 5-HT_7_ specific residue (at this position, other 5-HT receptors have an alanine, leucine, or threonine; see sequence alignment in Figure 4). A larger residue at this position could compromise the interaction between R180^3.50^ and L394^G.H5.26^. Consistent with this, the 3.53 position corresponds to the single nucleotide polymorphism G183^3.53^R, which is predicted to be deleterious (https://gpcrdb.org/protein/5ht7r_human/), underscoring the importance of this position.

**Figure 5:**
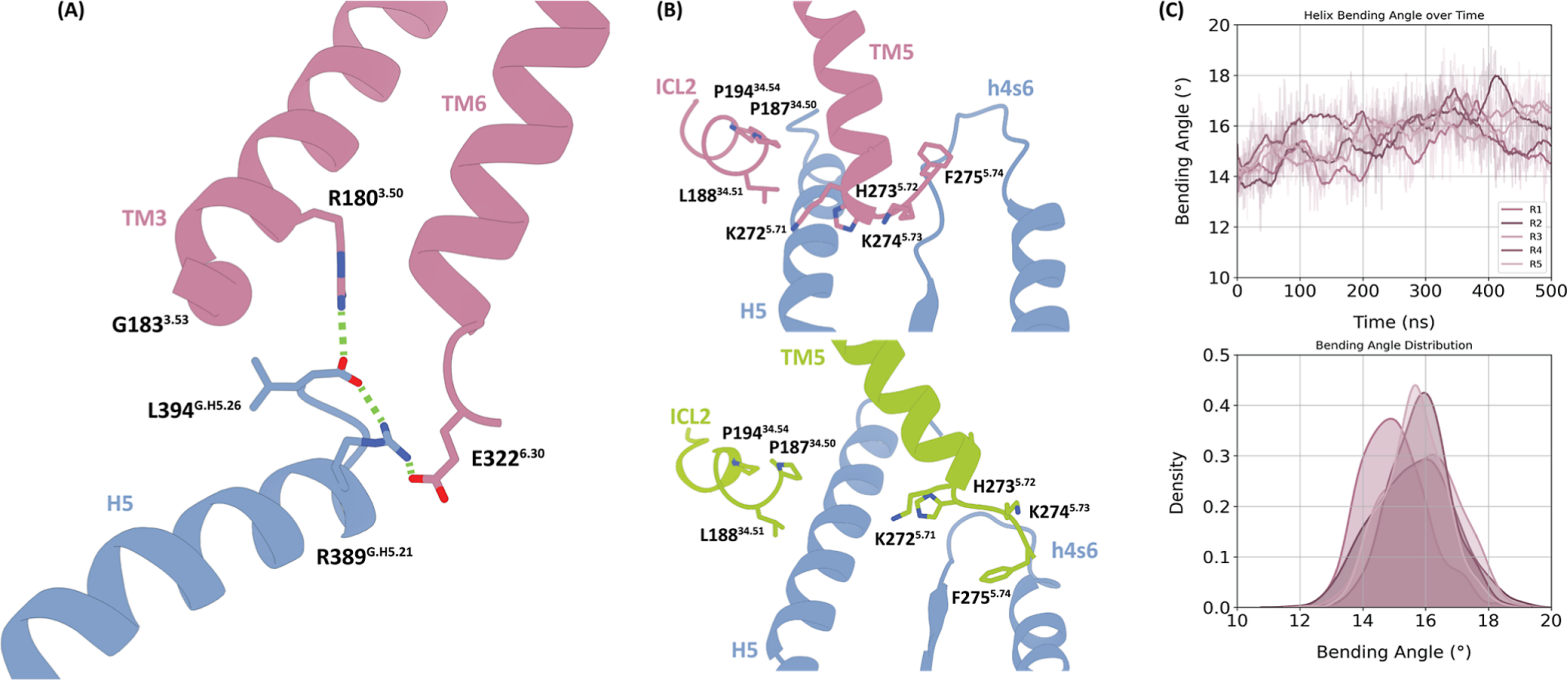
Molecular signature of the preassembled complex. (A) Interaction network between the conserved positions R180^3.50^ and E322^6.30^ of 5-HT_7_ with R389^G.H5.21^ and L394^G.H5.26^ in H5. Interacting residues are represented as stick and polar interactions with dashed green lines. (B) Structural representation of the interaction between the specific residues driving selectivity TM5 and ICL2 and the G_s_ in the preassembled (top) and active (bottom) complexes. (C) Time evolution and distribution of the bending angle of the TM5 in the preassembled complex.

The C-terminus of L394^G.H5.26^ establishes an intramolecular ionic lock with R385^G.H5.21^, so that these two residues link TM3 (R180^3.50^) and TM6 (E322^6.30^) together (Figure 5A). The significant role of the interaction between R389^G.H5.21^ and E322^6.30^ in the preassembled complex is in agreement with mutagenesis studies (Ballesteros, Jensen et al. 2001, Liu, Xu et al. 2019). Moreover, in the proposed binding mechanism of the β2-adrenergic receptor to the G_s_,^36^ R389^G.H5.21^ was found to engage in an ionic interaction with D130^3.49^, further supporting the relevance of this residue for the conformational rearrangement leading to the GPCR:G protein active complex.

In addition to interaction patterns involving positions that are conserved in all serotoninergic GPCRs (such as R180^3.50^ and E322^6.30^), our analysis also pinpoints key interactions involving 5-HT_7_-specific residues (Figure 4). Selective residues in ICL2 (e.g., P191^34.54^) and TM5 (e.g., F275^5.74^) are involved in interaction patterns specific to the active and preassembled states and, therefore, in our proposed activation mechanism (Figure 5B). Importantly, the lengths of TM5 and TM6 were found to vary among the resolved 5-HT receptors, suggesting the relevance of these regions to G protein selectivity.^29^

Residue K274^5.73^ has a stable interaction with Y358^G.h4s6.20^, a contact that is highly specific to the active complex. The entire terminal portion of TM5 (positions 5.71–5.74) forms an extensive interaction network with H5 and h4s6 of the G_s_ protein, serving to structurally link these domains (Figure 5B, bottom). These interactions are not present in the preassembled starting structure (Figure S8). However, during the simulations, TM5 shifts outward by approximately 16° (Figure 5D). This movement allows it to maintain anchoring interactions with h4s6, but not with H5, indicating a dynamic rearrangement that selectively stabilizes the preassembled complex (Figure 5B, top, Figure 4).

In the preassembled complex, ICL2 residues P187^34.50^, L188^34.51^, and P191^34.54^ are actively involved in interactions with H5 (P187^34.50^ with R385^G.H5.17^ and L388^G.H5.20^, L188^34.51^ with Q384^G.H5.16^, and P191^34.54^ with L388^G.H5.20^ and L393^G.H5.25^), showing over 50% interaction frequencies (Figure 4). However, the interaction network between ICL2 and H5 is broader in the active complex. Additional interactions are observed with residues at the first portion of H5 (F376^G.H5.08^, R380^G.H5.12^, and I383^G.H5.15^), as well as with residues from HN and S3, alongside Q384^G.H5.16^, H387^G.H5.19^, L388^G.H5.20^, and Y391^G.H5.23^. Previous studies on active GPCR:G_s_ complexes have shown that the movement of H5 toward ICL2 forms a hydrophobic cavity between the receptor and G protein.^37,38^ This cavity can accommodate bulky ICL2 residues and has been suggested to contribute to G_s_-coupling specificity.^29^ In the 5-HT_7_ receptor, this cavity specifically accommodates L188^34.51^. Notably, the presence of a conserved proline (P191^34.54^) in ICL2 can significantly impact the loop dynamics and the orientation of L188^34.51^, likely contributing to the stabilization of this interaction interface.

It should be noted that due to the uncertainty of ICL3 modeling, we excluded this loop from our analyses, despite the fact that it could play a key role in the activation mechanism.^29^ The uncertainty of ICL3 modeling^31,32,36,39–41^ and the possible variability in TM5 and TM6 length are indeed major challenges of this work, presenting a key area for future investigation. This will be addressed further as new experimental structures covering this area become available.

## 3 Conclusions

Using molecular dynamics simulations, we identified stable interaction patterns of the 5-HT_7_ receptor in complex with the G protein and highlighted unique structural features distinguishing active, inactive, and preassembled states of the receptor. Notably, the preassembled state reveals a distinct rearrangement of conserved residues R180^3.50^ and E322^6.30^, diverging from the orientation observed in the active and inactive states. This rearrangement supports a mechanistic hypothesis wherein the preassembled complex functions as an intermediate conformation that could facilitate the transition from the inactive to active conformation. Additionally, 5-HT_7_-specific ICL2 and TM5 residues appear to play a central role in stabilizing this preassembled architecture, offering new insights into receptor-specific modulation of signaling. Together, these findings propose a model of molecular mechanisms of 5-HT_7_ coupling with G_s_ and underscore the importance of receptor-subtype-specific elements in shaping GPCR signaling properties.

## 4 Methods

### 4.1 Modeling of the Inactive G_s_:GDP and Active G_s_

MODELLER^44^ (AutoModel) was used to reconstruct the missing residues in the GDP-bound G_s_ protein (PDB ID: 6EG8)­(positions 73–86). The active-state G_αs_ protein included mutations K274D, G226A, S54N, T284D, E268A, I285T, R280K, and N271K that were reversed to the wild type using the “Build” tool in Schrodinger. Missing residues (63–208, 253–259) were rebuilt with MODELER (AutoModel) using G_α_ structures in complex with dopamine and glucagon receptors (PDB IDs: 7JOZ and 6X18, respectively) as templates. Ten models were generated for both the G_s_:GDP state and the nucleotide-free state, and the best model was selected based on the DOPE score and visual inspection.

### 4.2 3D Structural Modeling of the 5-HT_7_ Inactive State and the 5-HT_7_:G_s_ Preassembled Complex

The sequence alignment between 5-HT_7_ (UniProtKB: P34969) and serotonin receptors was retrieved from GPCRdb Web server.^42,43^ The inactive state of 5-HT_7_ was generated based on the active-state experimental structure (PDB ID: 7XTC, resolution of 3.2 Å). The structures of 5-HT_2A_ (PDB ID: 7WC4, resolution of 3.2 Å) and 5-HT_1B_ (PDB ID: 4IAQ, resolution of 2.8 Å) were used as templates to model the regions involved in conformational changes: G77^1.28^–L123^2.46^, A325^6.33^–V338^6.46^, and N380^7.49^–Q402^8.61^. Twenty models were built with MODELER (AutoModel)^44^ (v10.2) and the best model was selected based on the DOPE score. For the regions of the 5-HT_7_ model where the template information was not available, i.e., residues F275^5.74^–K324^6.32^ and C354–C358 in the ECL3, ab initio modeling was performed using the *loopmodel class* implemented in MODELER.^45^ The cysteine residues forming the conserved TM3–ECL2 disulfide bridge^46^ (C155^3.25^–C231^45.50^), as well as C354 and C358 in the ECL3, were constrained to form a disulfide bond, as this is shown in the experimental structures of 5-HT_1a_ (PDB ID: 8W8B), 5-HT_1D_ (PDB ID: 7E32), 5-HT_2b_ (PDB ID: 5TUD), 5-HT_2c_ (PDB ID: 6BQH), and 5-HT_6_ (PDB ID: 8JLZ) receptors.

We performed loop refinement using both fast and slow algorithms. We built 1000 models for each of the protocols. All the models were aligned with the reference structure (PDB ID: 7E32) retrieved from the OPM database^47^ for the correct orientation with the membrane bilayer. We then selected the conformation with minimal clashes with the G_s_ protein and the membrane. We combined the modeled regions to the experimental structure using the protein splicer tool implemented in Maestro.^48^ The final model was minimized to a derivate convergence of 0.01 kJ/mol Å, the OPLS4 force field, and VSGB water solvation model, 65 steps per iteration, using the minimize tool in Bioluminate.^49–51^ We then manually changed the rotameric state of R180^3.50^ (similar to that of 6WH4.pdb) to ensure the formation of the ionic lock with E322^6.30^.

Figure S2 provides a representation of the regions modeled with the different approaches.

The preassembled complex was modeled following the approach of Mafi et al.^31^ We separately superimposed the inactive state model of 5-HT_7_ and inactive G_α_, G_β1_, and G_γ2_ to corresponding protein chains in the active state 5-HT_7_:G_s_ complex using the protein structure alignment tool available in Maestro.^48^

### 4.3 System Preparation of Preassembled and Active Complexes

The preassembled and the active state complexes were prepared using the protein preparation wizard^52^ implemented in Maestro.^48^ During this protein preparation, the bond orders were assigned, hydrogens were added, and disulfide bonds were created with Epik at pH 7.4 ± 2.0.^53^ The hydrogen-bond network of the complexes was optimized with PROPKA,^54^ proper protonation state for the optimization of His, Glu, and Asp was monitored using the Interactive optimizer. The residue D127^2.50^ was maintained neutral in the active-state complex and deprotonated for the preassembled complex.^25^ Minimization of the hydrogens was performed using the OPLS4 Force Field.^55^

GetContacts (https://getcontacts.github.io/) was used for analysis of the interaction interface between 5-HT_7_ and the G_α_ protein in the active-state complex and the preassembled complex in the initial static structures. By using the get_static_contacts.py tool, we created a list of all the interacting residues at the interface (within 4.5 Å distance). The interface of the preassembled complex involves ICL1 (109^1.60^–116^2.39^), TM3 (152^3.22^–185^3.55^), ICL2 (186^3.56^–195^4.38^), TM5 (237^5.36^–272^5.71^), ICL3 (273^5.72^–320^6.28^), TM6 (321^6.29^–349^6.57^), and H8 (389^8.48^–402^8.61^) of 5-HT_7_, and αN, α5, α4, β1, β2, β6, and H4S6 regions of the G_α_ protein. Next, using the “*get_contact_frequencies*.*py*” module, we calculated the frequency of each interaction found in the contact list such as polar interactions (hydrogen bonds, salt bridges) and nonpolar interactions (Hydrophobic, π-stacking, T-stacking, and π-cation).

Homolwat Web server^56^ was used to add water molecules within the receptor structures, applying settings described in the GPCRmd protocol.^25^ The orientation of the prepared complexes within the membrane bilayer was obtained from the coordinates of the 5-HT_1D_ receptor (PDB ID: 7E32), as deposited in the Orientations of Proteins in Membranes (OPM) database.^57^ The two complexes were superimposed on the X-ray irradiation of the OPM reference structure. The prepared complexes were then embedded into a prebuilt (with VMD Membrane Builder plugin 1.1) 1-palmitoyl-2oleyl-*sn*-glycerol-3-phospho-choline (POPC) square bilayer of 131 Å × 131 Å × 165 Å and 143 Å × 143 Å × 160 Å for active and preassembled complexes, respectively, through an insertion method^58^ by using HTMD^59^ (Acellera, version 2.0.8). Lipids overlapping with protein residues were removed. TIP3P water molecules were added to the simulation boxes by using VMD Solvate plugin 1.5. The overall charge neutrality was maintained by adding Na^+^/Cl^–^ ions to reach a final physiological concentration of 0.154 M by using VMD Autonize plugin 1.3. All the N- and C-terminus chains (GPCR, G_α_, G_β_, G_γ_) were capped with ACE and CT3, with the exception of G_α_ helix 5 (L394^H5.26^), which remained negatively charged. This preparation protocol was also applied for the system preparation of the 5-HT_7_ inactive state (with full ICL3) without the heterotrimeric G protein. The difference relies on the membrane size. Here, we embedded the inactive GPCR into a 1-palmitoyl-2oleyl-*sn*-glycerol-3-phospho-choline (POPC) square bilayer of 120 Å × 120 Å. TIP3P water molecules were added to the 116 Å × 116 Å × 126 Å simulation box with the same concentration of counterions.

### 4.4 MD Simulation Protocol and Analyses

The CgenFF^60^ (v4.6) and CHARMM36 ^61,62^ for protein, lipid, TIP3P water model, GDP, and nucleic acid were used for this work. The topology and parameters of the cocrystallized ligand (5-Carboxamidotryptamine, 5-CT) in 7XTC were obtained from the ParamChem Web server (https://cgenff.umaryland.edu/). We simulated three systems: 5-CT:5-HT_7_:G_s_, preassembled complex 5-HT_7_:G_s_:GDP, and 5-HT_7_ inactive state without the G-protein (Table S1).

ACEMD^63^ (Acellera, version 3.5.1) was used for MD simulations with periodic boundary conditions. The systems were initially equilibrated through a 5000 conjugate gradient step minimization to reduce clashes induced by the system preparation between protein and lipid/water atoms and then equilibrated with 120 ns MD simulation in the isothermal–isobaric conditions (*NPT* ensemble), employing an integration step of 2 fs. The temperature was maintained at 310 K using a Langevin thermostat^64^ with a low damping constant of 1 ps^–1^, and the pressure was maintained at 1.01325 atm using a Monte Carlo barostat. Initial restraints of 5 kcal mol^–1^ Å^–2^ were gradually reduced in a multistage procedure over the 120 ns: 6 ns for lipid phosphorus atoms, 90 ns for all protein atoms other than C_α_ atoms, 100 ns for the protein C_α_ atoms, and 120 ns for GDP or cocrystallized ligand. The M-SHAKE algorithm^65^ was used to constrain the bond lengths involving hydrogen atoms. Long-range Columbic interactions were handled using the particle mesh Ewald summation method^66^ with a grid size rounded to the approximate integer value of cell wall dimensions. The cutoff distance for long-term interactions was set at 9.0 Å, with a switching function of 7.5 Å.

To evaluate the stability and the biophysical validity of the equilibrated systems, the average area per lipid (ApL) headgroup with VTMC,^67^ the bilayer thickness with MEMPLUGIN,^68^ and the volume of the simulation box were calculated. The computed ApL and thickness were in agreement with the experimental values measured for the POPC lipid bilayers. We run five independent replicas for each equilibrated system of 500 ns unrestrained MD simulations in the canonical ensemble (*NVT*) with an integration time step of 4 fs. The temperature was set at 310 K, by setting the damping constant at 0.1 ps^–1^.

RMSD and RMSF of the backbone carbon alpha were computed for each chain (GPCR, G_α_, G_β_, G_γ_) with an in-house python script based on MDAnalysis (v2.2.0).^69^ We used as a reference the starting structures. We computed the RMSD using two types of alignment: first, chain-to-chain alignment (the ICL3 region of 5-HT_7_ and G_α_ residue 64–87 were excluded from the alignment); second, we used as a reference the GPCR carbon alpha atoms excluding the ICL3 region. To compute the RMSF, we used chain-to-chain alignment. The 5-HT_7_ active structure was used as a reference for both aligning and computing the RMSD values of R385^G.H5.17^ and Y391^G.H5.21^.

For the analysis of the interactions of the MD simulations, the five replicas for each system were merged into a single trajectory. Given the uncertainty in modeling the ICL3 region, we selected a conformation that did not interfere with the G protein. These rebuilt residues were excluded from subsequent analyses. MDciao python module (v0.5)^70^ (https://github.com/gph82/mdciao) was used to calculate and compute the interaction frequencies between the GPCR and the G_α_. We set the cutoff to 4 Å and computed a number of maximum contacts of 80, excluding the first 100 ns of each replica. The distance of the salt bridge between residues R180^3.50^ and E322^6.30^ was monitored using the distance between the CG atom of E322^3.50^ and the CZ atom of R180^3.50^, calculated with the module “*distances*.*distance_array*” in MDAnalysis (v2.2.0).^69^

To evaluate conformational changes in TM5 across different replicas of the preassembled complex, we computed the bending angle (θ) from the positions of C_α_ in two consecutive residue segments along TM5. We define two vectors: (i) Vector 1 ( ${\mathrm{v⃗}}_{1}$ ): from the first to the last C_α_ atom of residues F237^5.36^–Y249^5.48^ (extracellular portion), (ii) Vector 2 ( ${\mathrm{v⃗}}_{2}$ ): from the first to the last C_α_ atom of residues I250^5.49^–H273^5.72^ (intracellular portion). The bending angle θ at each simulation frame was computed by using the dot product of the normalized vectors:

1 $$\theta =\frac{{\mathrm{v⃗}}_{1}\cdot {\mathrm{v⃗}}_{2}}{∥{\mathrm{v⃗}}_{1}∥∥{\mathrm{v⃗}}_{2}∥}$$

where the ${\mathrm{v⃗}}_{1}\cdot {\mathrm{v⃗}}_{2}$ denotes the dot product and $∥{\mathrm{v⃗}}_{i}∥$ is the Euclidean norm of ${\mathrm{v⃗}}_{i}$ . The calculation was computed across every frame of each trajectory using MDAnalysis (v2.2.0).^69^ Rendering of the structural images was done with ChimeraX (v1.9).^71^ Visualization of all data was done with the Matplotlib and seaborn Python library.^72,73^

## Supplementary Material



## Data Availability

Topology, parameter, and coordinate files as well as MD trajectories are available at https://zenodo.org/records/15195899. The MD simulation of system 1 have been deposited into the GPCRmd database (https://www.gpcrmd.org/) under access codes 2370 (https://www.gpcrmd.org/view/2370/).

## Notes

Heterotrimeric G_s_ protein representations, representation of the 5-HT_7_ refined models, electrostatic surface of 5HT_7_:G_s_ complexes, RMSD and RMSF analyses, H5 conformational space, and distance plot of ionic lock (PDF)
