# Supplementary material for: Conformational Signatures of Preassembled and Active Complexes of 5‑HT7 with the Gs Protein
Source: J Chem Inf Model. 2025 Oct 24;65(21):11826–36. doi: 10.1021/acs.jcim.5c01698 (PMC12606645; doi:10.1021/acs.jcim.5c01698)
Supplement: Supplementary file 1 [file ci5c01698_si_001.pdf]

Supporting Information

Conformational Signatures of Preassembled and Active Complexes of 5-HT_7 with the G_s Protein

Zeenat Zara,[1,2,#] Alessandro Nicoli,[1,3,#,*] Ruiming He,[1,4] Natalia Kulik,[5] David Reha,[6] Alexey Bondar,[2,7] Antonella Di Pizio[1,3,*]

[1]Leibniz Institute for Food Systems Biology at the Technical University of Munich, 85354 Freising, Germany

[2]Faculty of Science, University of South Bohemia in Ceske Budejovice, Branisovska 1760, 370 05 Ceske Budejovice Czech Republic

[3]Professorship for Chemoinformatics and Protein Modelling, TUM School of Life Sciences, Technical University of Munich, 85354 Freising, Germany

[4]Center for Functional Protein Assemblies (CPA), Department Bioscience, TUM School of Natural Science, Technical University of Munich, Ernst-Otto-Fischer-Strasse 8, Garching, Germany.

[5]Laboratory of Photosynthesis, Centre Algatech, Institute of Microbiology of the Czech Academy of Sciences, Novohradská 237, Třeboň CZ-37981, Czech Republic

[6]IT4Innovations, VSB – Technical University of Ostrava, 17. listopadu 2172/15, 708 00 Ostrava-Poruba, Czech Republic

[7]Institute of Plant Molecular Biology, Biology Centre of the Czech Academy of Sciences, Branisovska 1160/31, 370 05 Ceske Budejovice, Czech Republic

[#]equal contribution

* Correspondence:

a.dipizio.leibniz-lsb@tum.de, Tel.: +498161716516

a.nicoli.leibniz-lsb@tum.de, Tel.: +498161716517

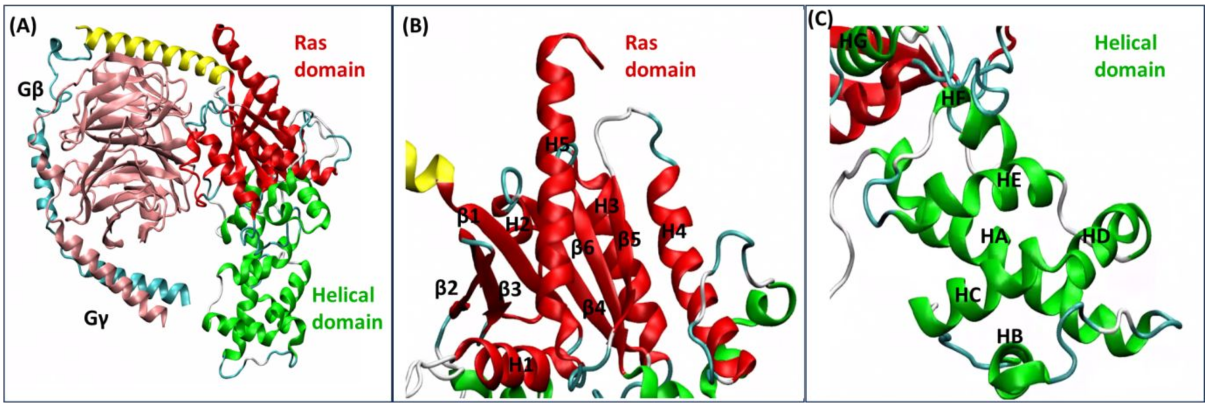

Figure S1. Heterotrimeric Gs protein and subdomains. (A) Ribbon model of heterotrimeric Gs protein. The Gα Ras domain (RD) and the Gα helical domain (HD) are colored in red and green cartoon, respectively. The Gβ and Gγ are depicted as light pink and light blue cartoon. (B) Zoom-in view of β1-6 and H1-H5 of the RD. (C) Zoom-in view of the HA-HF of the HD.

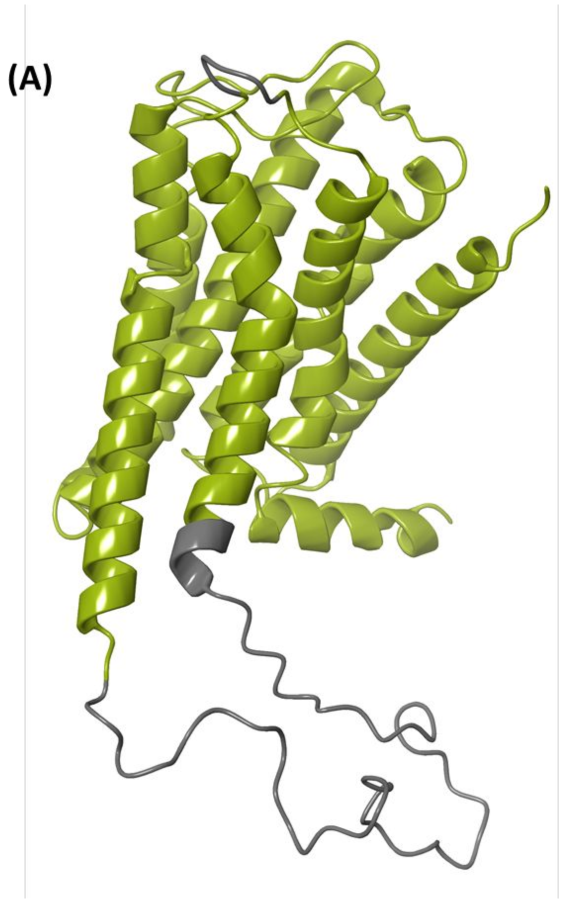

Figure S2. Active (A) and inactive (B) 5-HT$_7$ structures. (A) Refined model of the 5-HT$_7$ structure in the active state. (B) 5-HT$_7$ model in the inactive state. The regions used from the active state structure (PDB ID: 7XTC) are shown in green. The regions modeled from templates, 5-HT$_{2a}$ and 5-HT$_{1b}$ (G77$^{1.28}$-L123$^{2.46}$, A325$^{6.33}$-V338$^{6.46}$, N380$^{7.49}$-Q402$^{8.61}$) are colored in purple, and the *ab initio* regions (ECL3 and ICL3) in gray.

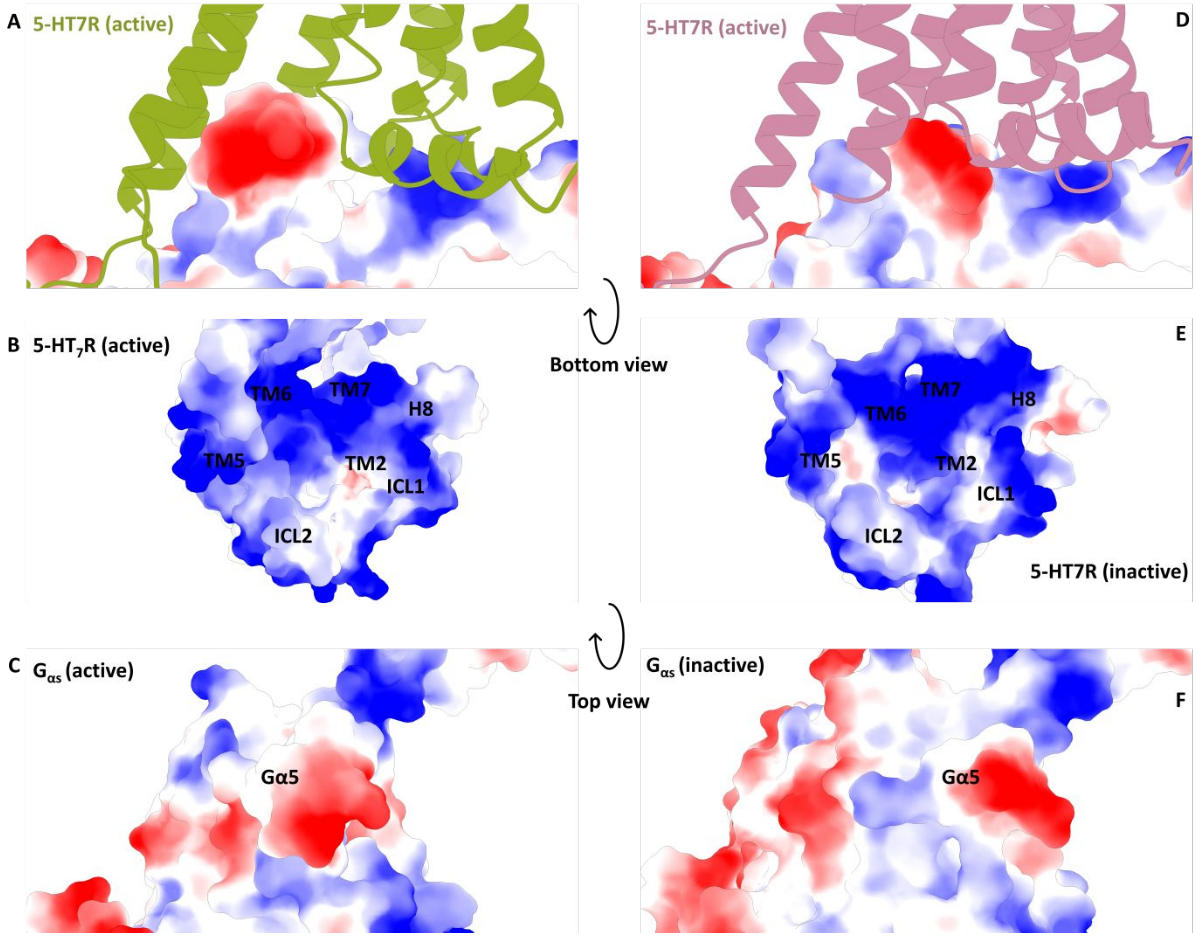

Figure S3. Electrostatic surface representation of the interaction interface between 5-HT7 and Gs protein in the fully active complex (A) and the preassembled complex (D). Panels (B–E) show the intracellular cavity of the G protein, while panels (C–F) highlight the surface of the α5 helix (H5).

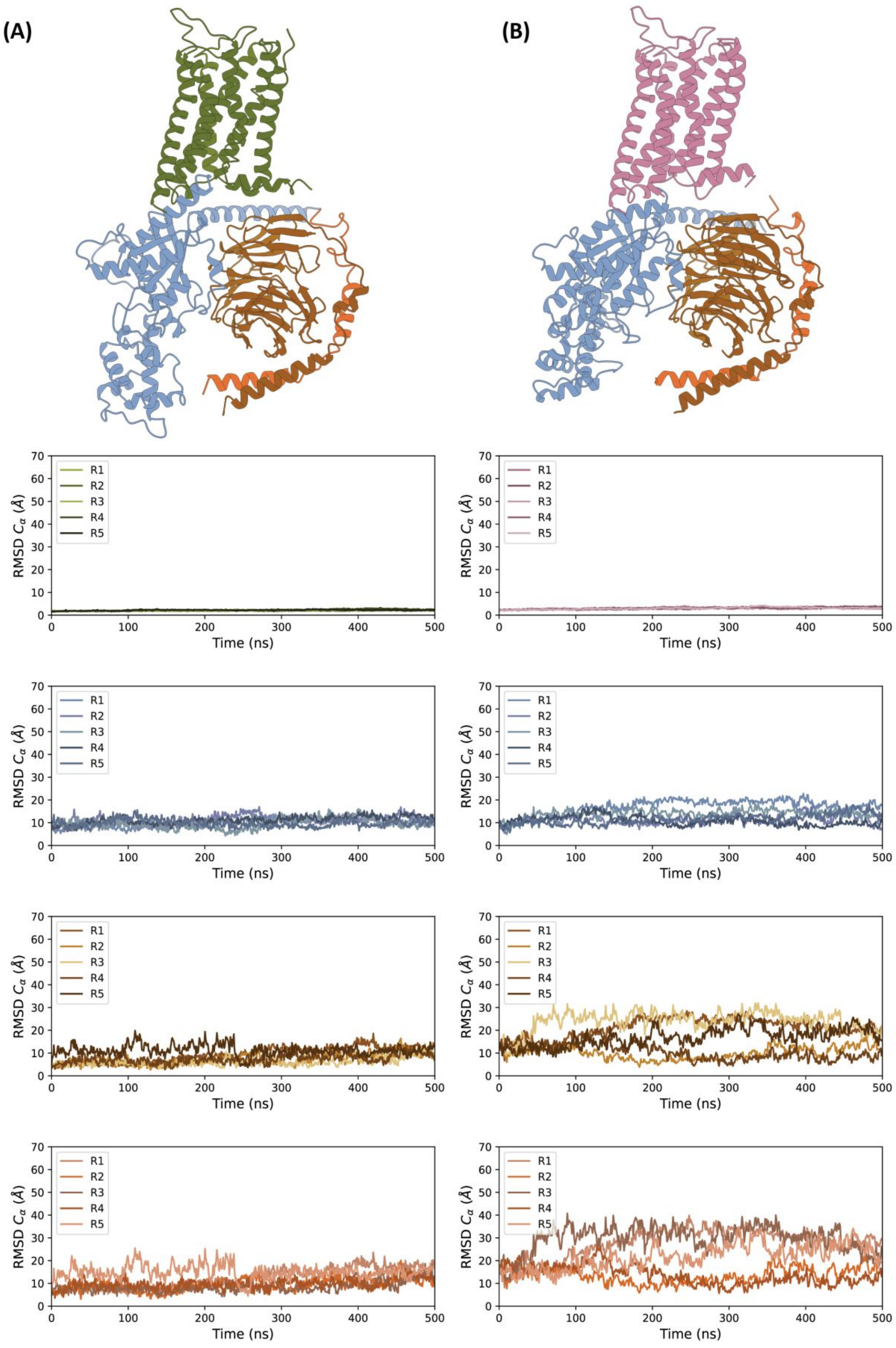

Figure S4. Root mean square deviation (RMSD) plots for active and inactive 5-HT$_7$ complexes with Gs during simulation (GPCRs alignment). The carbon alpha atoms of 5-HT$_7$ (excluding the ICL3 region) were used as the reference for the structural alignment. At the top is the structural representation of the simulated complexes, where the active state 5-HT$_7$ is depicted in olive green and the inactive in deep pink carton. The heterotrimeric protein is depicted as blue, brown and orange for Gα, Gβ and Gγ.

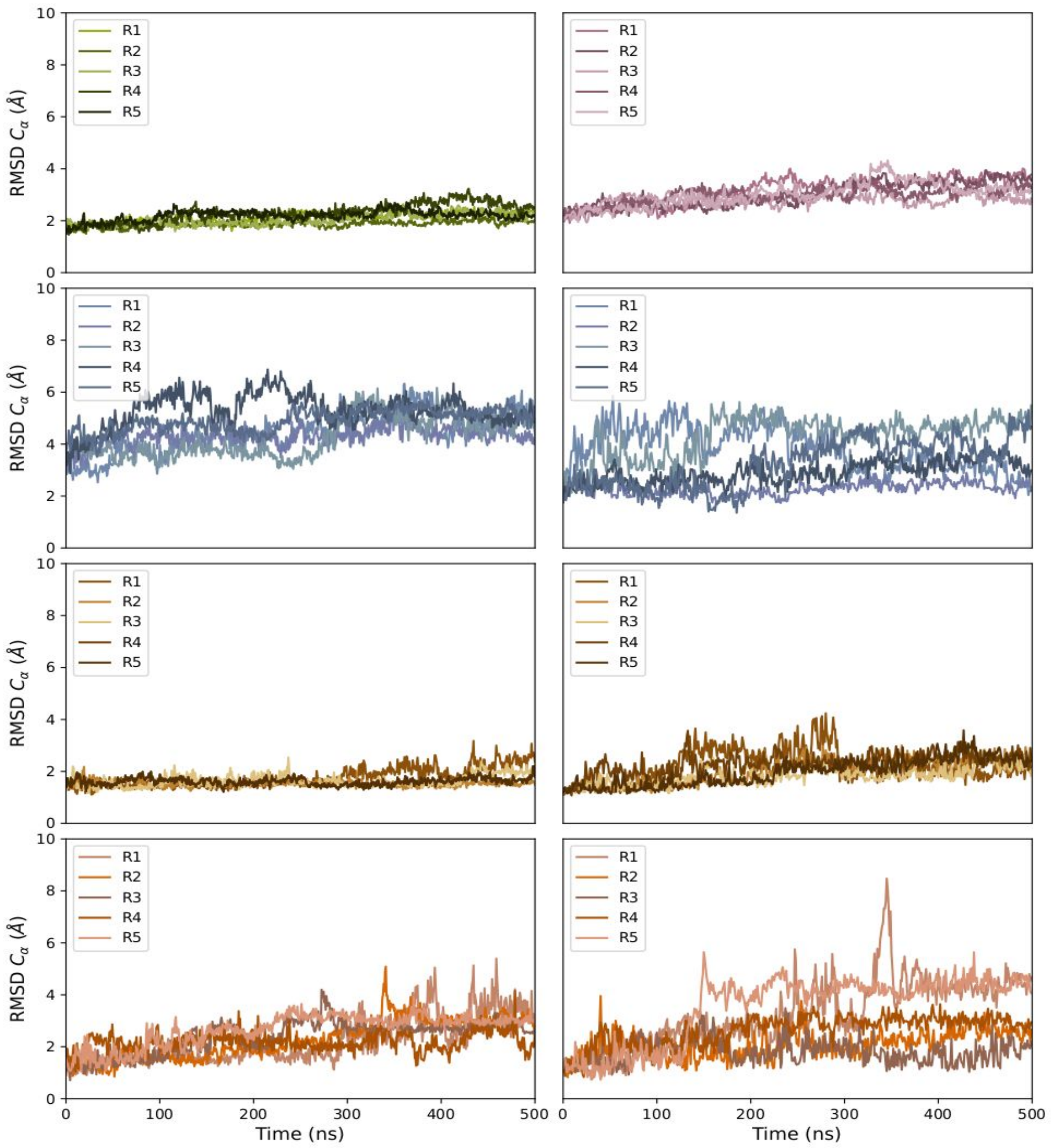

Figure S5. Root mean square deviation (RMSD) plots for active and inactive 5-HT$_7$ complexes with G$_s$ during simulation (Chain vs Chain). RMSD plots obtained on the alignment chain *vs.* the initial coordinates for active and preassembled complexes. We found no significant variation in RMSD values of the 5HT$_7$ in the preassembly and coupling complexes. There is a slightly increasing value of RMSD after 250ns for the 5-HT$_7$ in the preassembly complex due to the insertion of the G-protein within its binding site. The G$_\alpha$ subunit displayed the highest overall RMSD among all the other protein partners. Interestingly, the G$_\alpha$ subunit in the active complex showed a slightly higher RMSD than in the preassembled ones. Instead, G$_\beta$ is relatively stable in both complexes. The G$_\gamma$ has a relatively similar value of RMSD between the two systems, with the exception of R1 of preassembly complex, which is stable for the first part of the simulations (to 250ns), but it consistently deviated more in the second part.

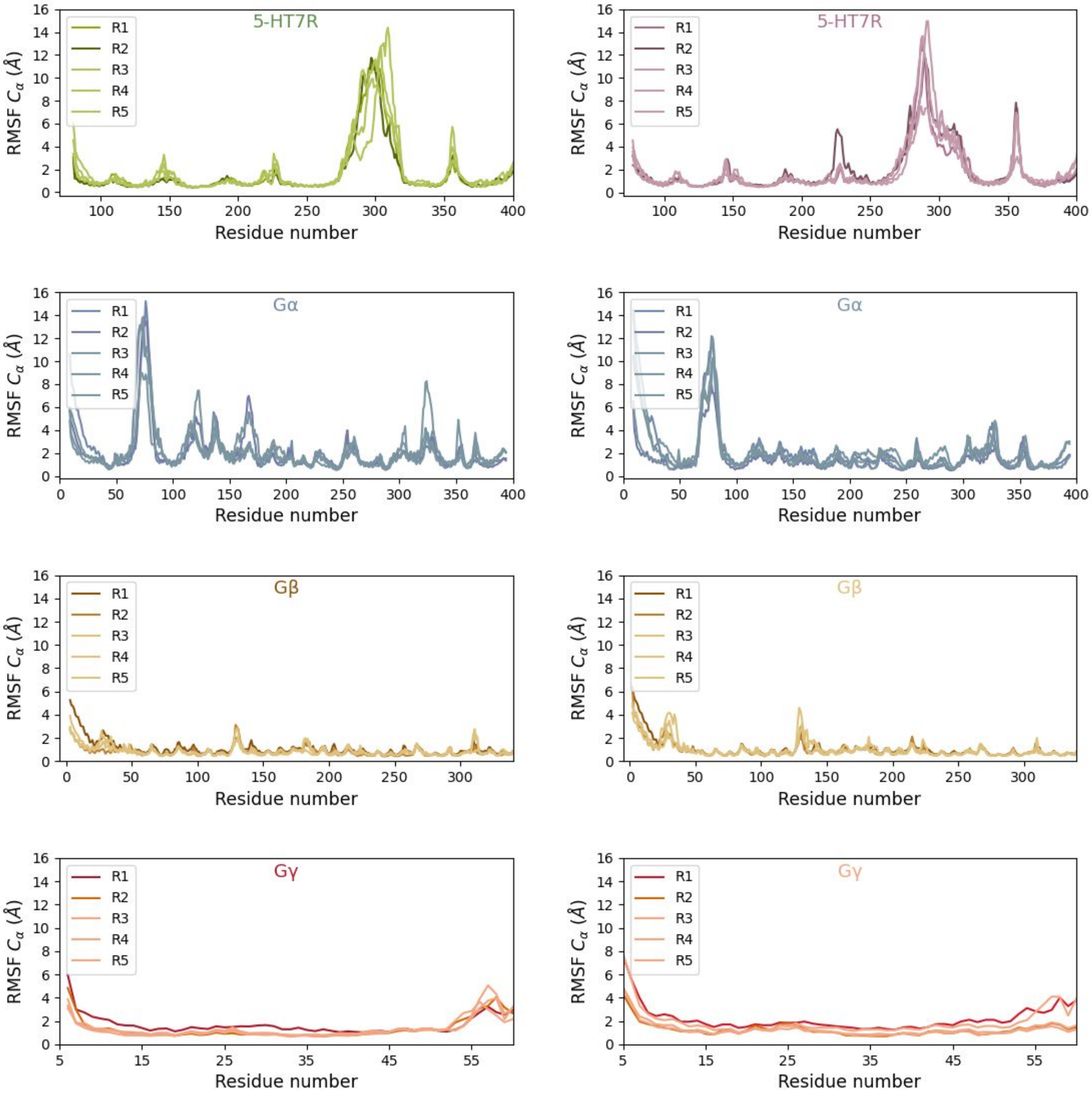

Figure S6. Root mean square fluctuation (RMSF) plots for the MD simulations of the active and preassembled 5-HT$_7$ complexes with G$_s$. RMSF plots for chain by chain for active and inactive complexes. The highest RMSF values were found for the unstructured ICL3 (F275$^{5.74}$-K324$^{6.32}$) of 5-HT$_7$ and HD of G$_\alpha$.

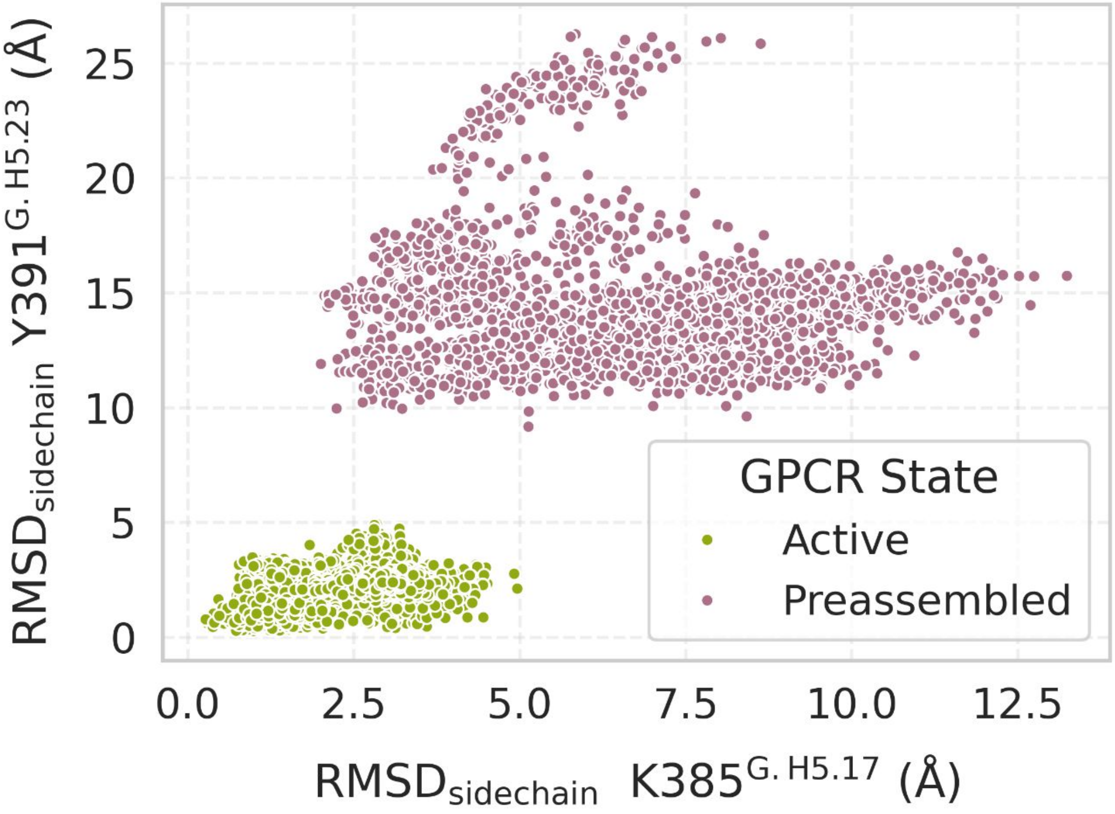

Figure S7. RMSD distributions of K385$^{G.H5.17}$ *vs.* Y391$^{G.H5.23}$ in the G_s active and preassembled complex. The carbon alpha atoms of the active 5-HT$_7$ (excluding the ICL3 region) was used as a reference structure for the alignment of both systems. Green and pink dots represent the RMSD values of active and preassembled.

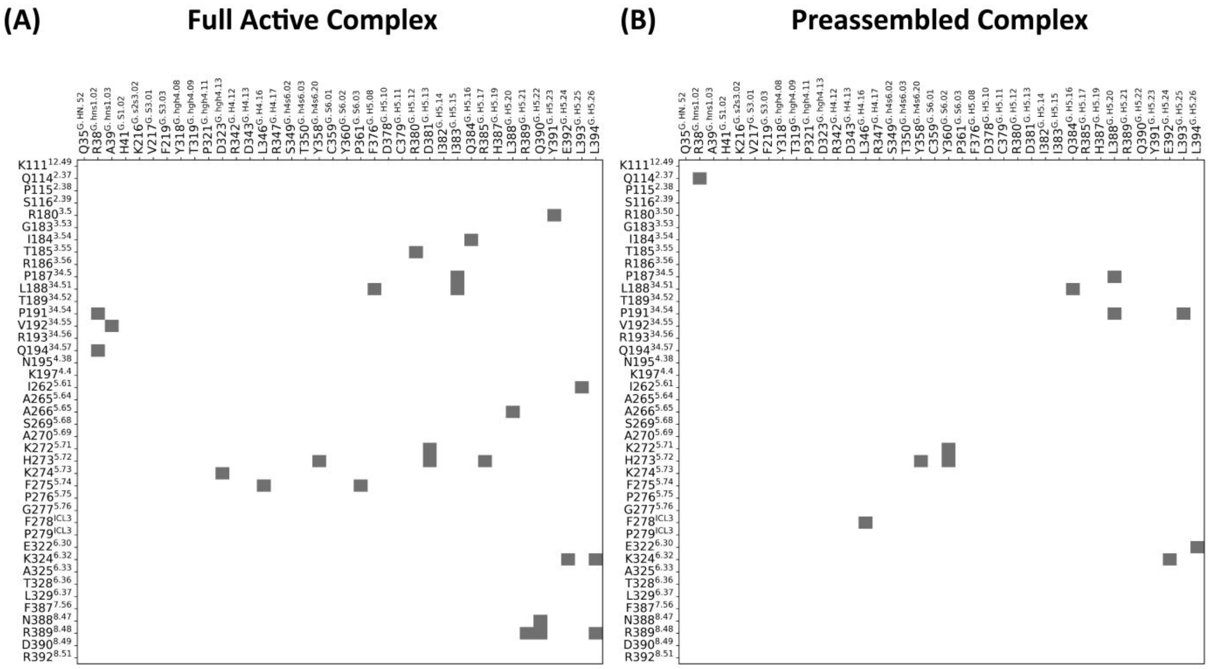

Figure S8. Interaction profiles of the active and preassembled states in their initial static structures (before MD simulations).

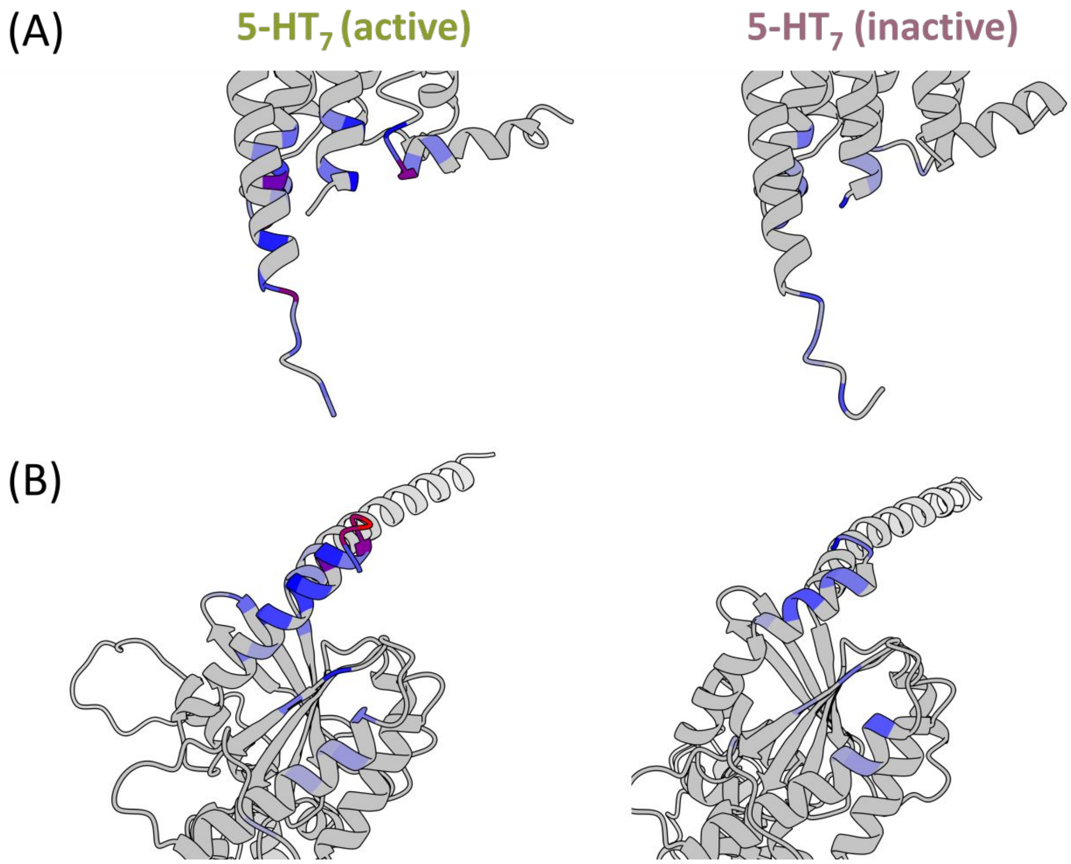

Figure S9. Projection of interaction frequencies on the 3D structural models of 5-HT$_7$ (A) and G$_\alpha$ (B) in the active (on the left) and preassembled (on the right) complexes. 5-HT$_7$ and G$_\alpha$ are represented as cartoons. The interactions are color-coded in shades of blue to red. Grey colored regions have zero contacts.

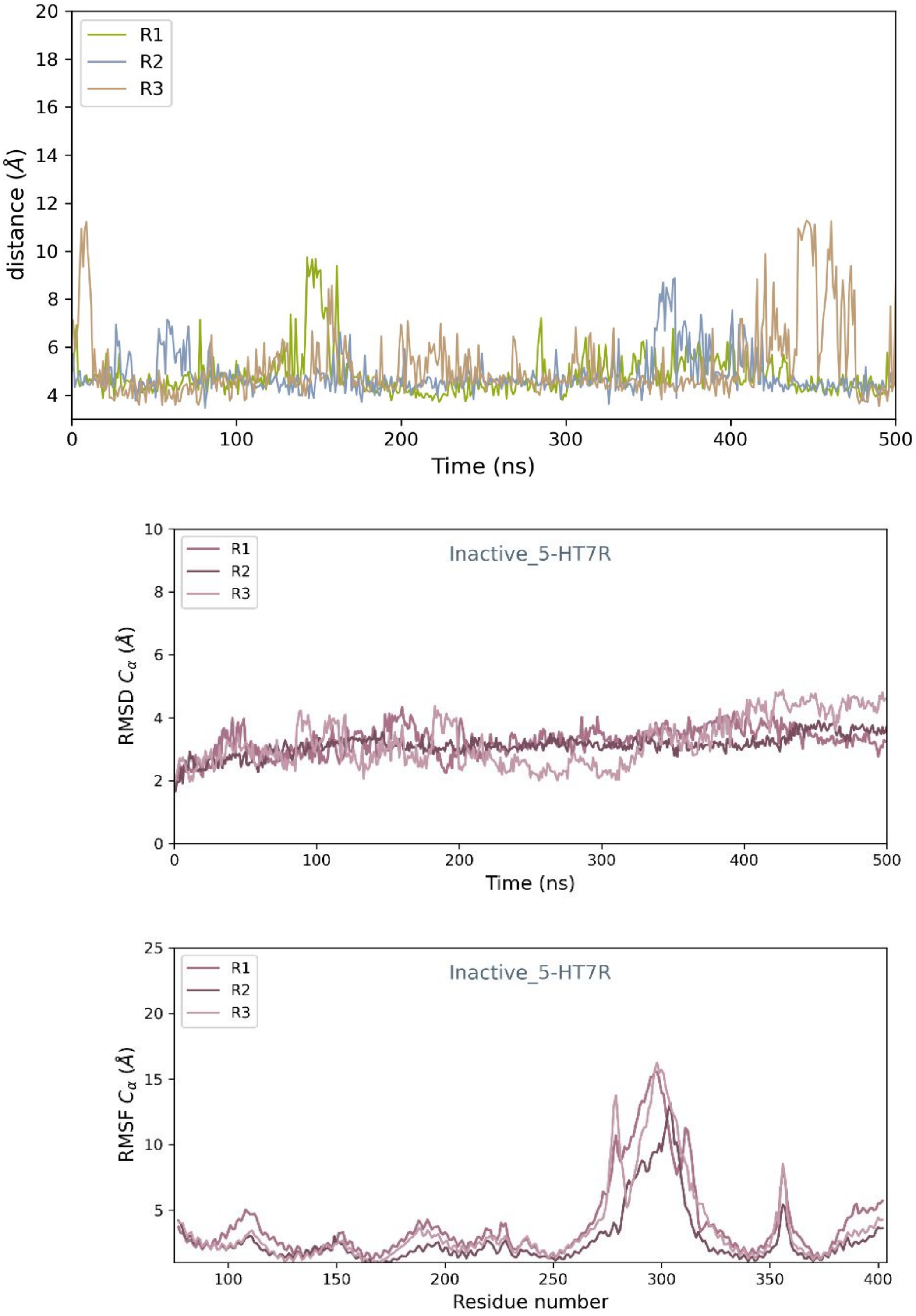

Figure S10. RMSD (top) and RMSF (bottom) plots for the MD simulations of the apo 5-HT$_7$ inactive state conformation without the G$_\alpha$ protein. The most apparent difference between the apo and inactive complex relies on the ICL3 flexibility, which is more flexible in the apo state as compared to preassembly complex because of the stabilizing effect of the G protein in the complexes.

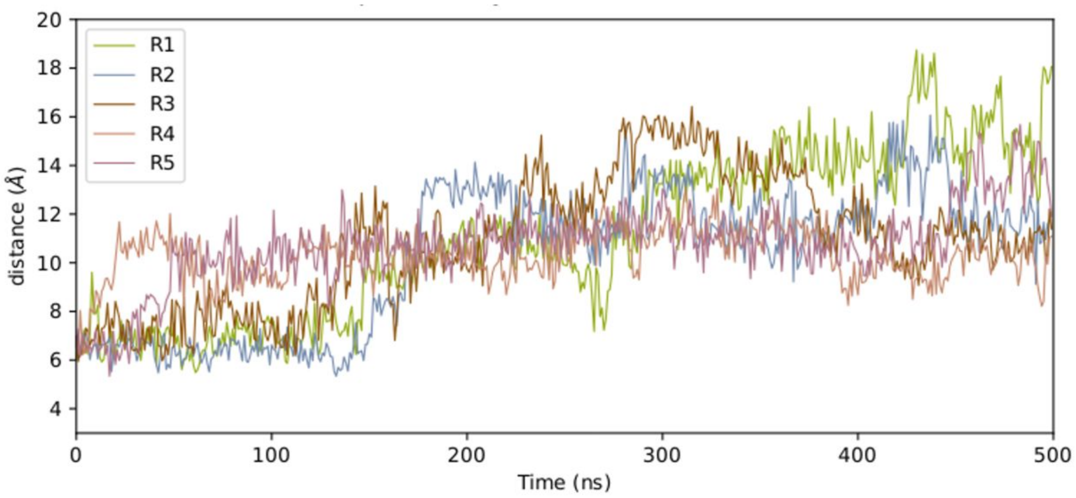

Figure S11. Distance between the residues R180$^{3.50}$ and E322$^{6.30}$ in the MD simulations of the apo 5-HT$_7$ in the preassembled complex (bottom).

Table S1. List and information of simulated systems. MD trajectories and related files (topology, parameter, and coordinates) are available at the Zenodo repository: https://zenodo.org/records/15195899.

System	Name	State	Ligand	Type	# atoms	Length (µs)	Repeats	Total simulated time (µs)
1	5-CT:5-HT$_7$:G$_s$	Active	5-CT	8F76	273350	0.5	5	2.5
2	5-HT$_7$:G$_s$:GDP	Inactive	GDP	Model	287296	0.5	5	2.5
3	5-HT$_7$	Inactive	APO	Model	163068	0.5	3	1.5
